# Uncommon cause of cholangitis due to a migrated pancreatic stone into the common bile duct

**DOI:** 10.1016/j.vgie.2020.07.012

**Published:** 2020-08-18

**Authors:** David M. de Jong, Jan W. Poley, Marco J. Bruno, Pieter J.F. de Jonge

**Affiliations:** Department of Gastroenterology and Hepatology, Erasmus MC University Medical Center, Rotterdam, the Netherlands

**Keywords:** CBD, common bile duct, MPD, main pancreatic duct

A 62-year-old man was referred to our tertiary hospital for endoscopic therapy of previously diagnosed alcoholic chronic pancreatitis. In the previous year he had been admitted to the referring hospital every 2 months for flares of pancreatitis, which were all treated conservatively. He was using nonopioid analgesics. He had no history of a cholecystectomy. Routine laboratory tests did not show any abnormalities. Cross-sectional imaging studies demonstrated 2 obstructive pancreatic duct stones in the pancreatic head with upstream main pancreatic duct dilatation of 7.6 mm. In addition, a 3.3- × 1.7-cm cyst was located at the pancreatic head in apparent communication with the pancreatic duct (Fig. 1).

**Figure 1:**
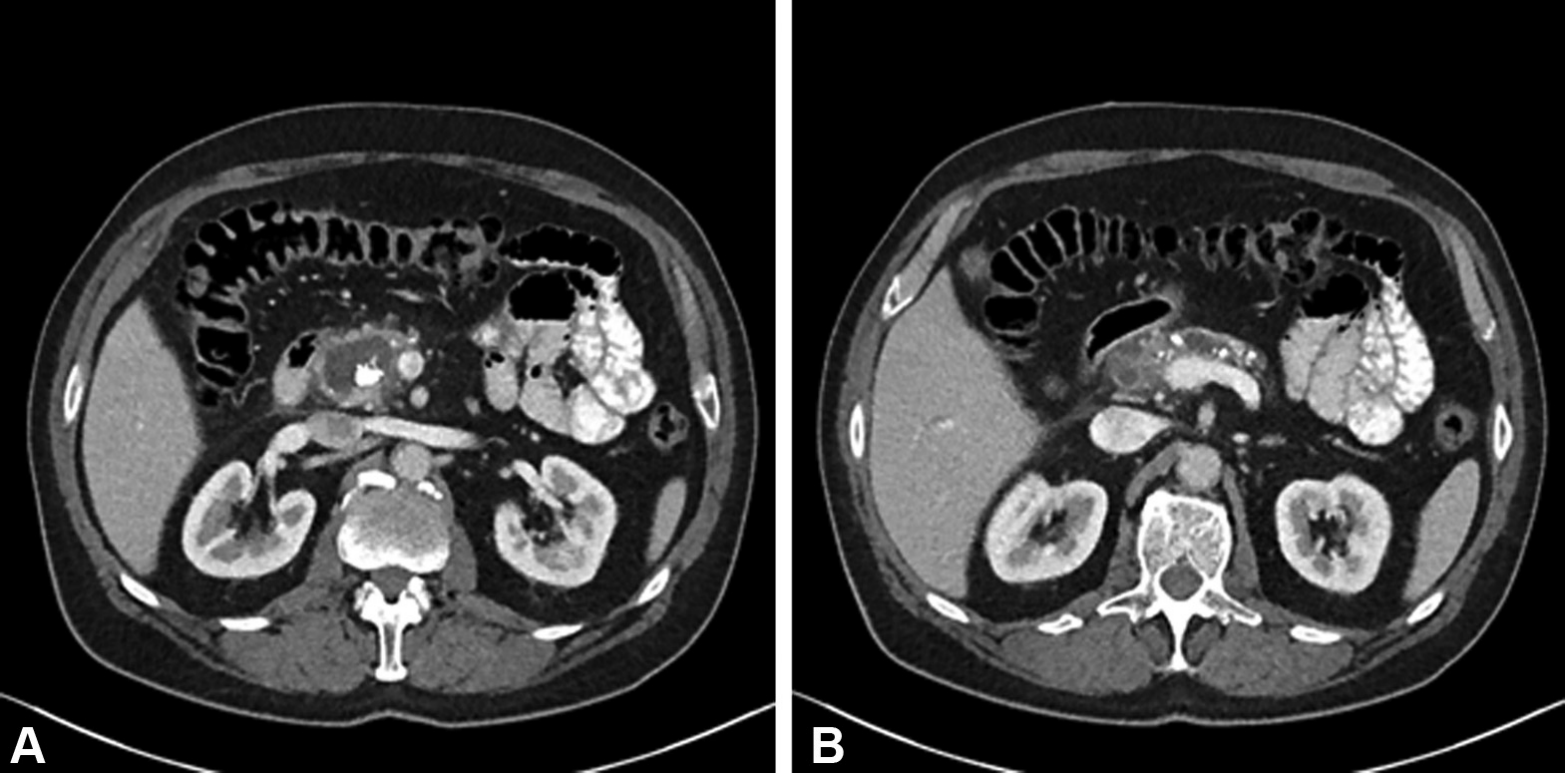
**A andB**: CT of the abdomen shows two obstructive pancreatic duct stones with upstream dilatation of the main pancreatic duct. Image A shows a pseudocyst in communication with the pancreatic duct.

The patient was scheduled for ERCP with pancreatic duct stent placement and, in a second stage, electrohydraulic lithotripsy of the pancreatic duct stones. On first ERCP, fluoroscopy demonstrated a duct stricture located in the pancreatic head, with intraductal calculi and upstream dilatation of main pancreatic duct (MPD) with opacification of the pseudocyst. The stricture was balloon dilated to 4 mm, and a 7F stent was inserted in the pancreatic duct (Fig. 2). The procedure was complicated by a mild post-ERCP pancreatitis.

**Figure 2:**
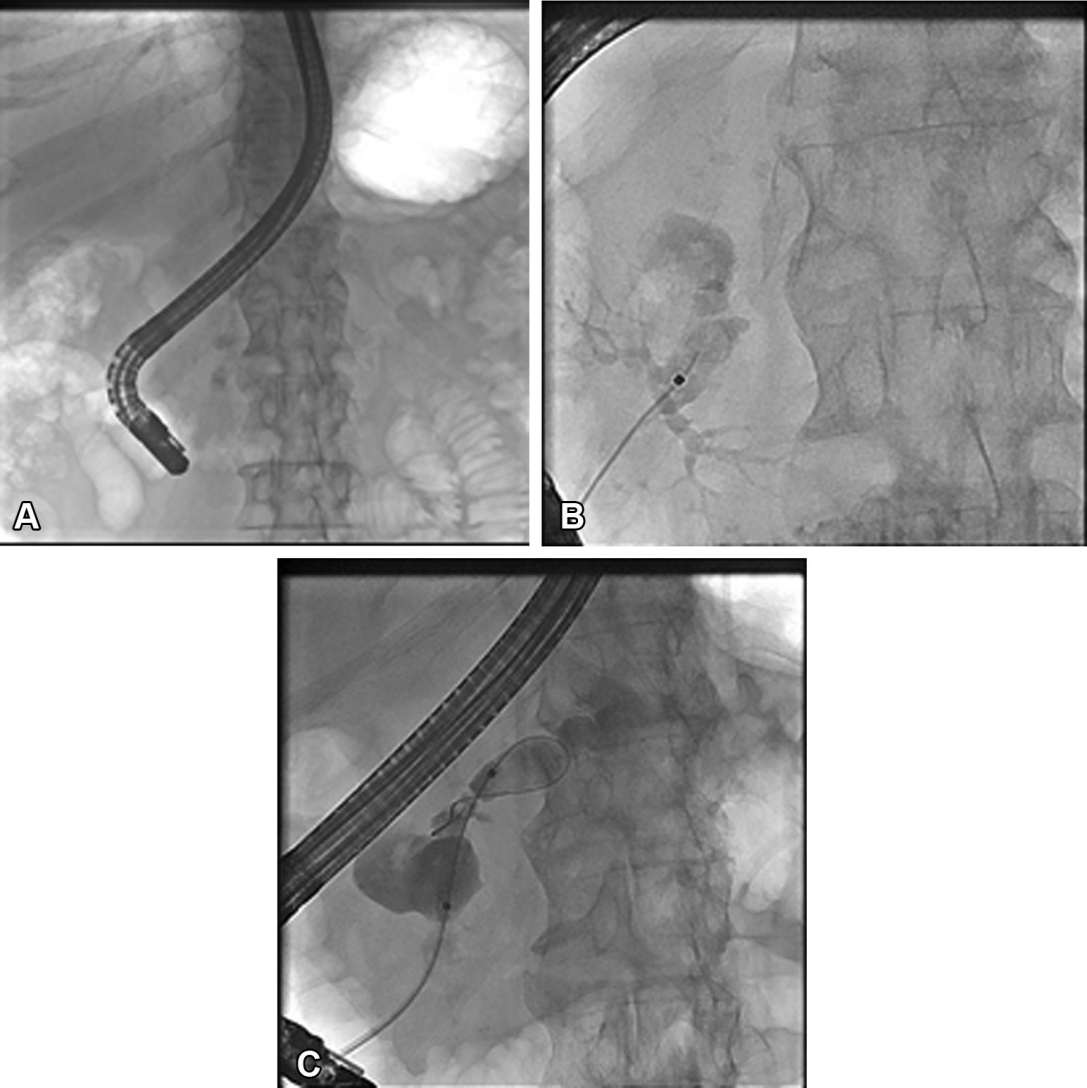
**A, B, andC**: Fluoroscopic images demonstrating a duct stricture in the pancreatic head with intra-ductal calculi and the pseudocyst.

Two months later, after outpatient evaluation confirmed clinical improvement, a second ERCP was scheduled for electrohydraulic lithotripsy of pancreatic duct stones. Fluoroscopy revealed resolution of the pseudocyst. Surprisingly, backflow of contrast into the bile duct was noted, strongly suggestive for a pancreaticobiliary fistula. Two large pancreatic stones measuring 8 to 9 mm were located in the Wirsung duct and probably the Santorini duct, respectively. A major portion of the stone in the Wirsung duct was treated with Spyglass electrohydraulic lithotripsy (SpyGlass® Direct Visualization System, Boston Scientific Corp., Natick, Mass). The orifice of the fistula could not be clearly identified on pancreatoscopy. A new pancreatic stent was deployed in the pancreatic duct.

Two months later, the patient was hospitalized in the referring hospital for acute cholangitis. A CT scan showed compression of the bile duct by the pancreatic stone (Fig. 3). Urgent ERCP was performed, and a biliary stent was placed. The patient was transferred to our hospital for further treatment. Fluoroscopy revealed a calcified stone in the common bile duct (CBD) which could not be extracted by a balloon catheter, as it was difficult to mobilize the stone within a relatively tight CBD. A Spyglass cholangioscopy was performed (Video 1, available online at www.VideoGIE.org). In the distal bile duct, a large pancreatic stone was seen; this was fragmented by electrohydraulic lithotripsy. The stone fragments were removed with an extraction balloon. Afterward, the residual stone in the pancreatic duct was fragmented as well. After contrast injection, a pancreatic fistula could not be demonstrated. We hypothesize that the stone migrated into the CBD via a fistula from the Santorini duct after the second ERCP. After 4 months of follow-up, the patient is still doing well.

**Figure 3:**
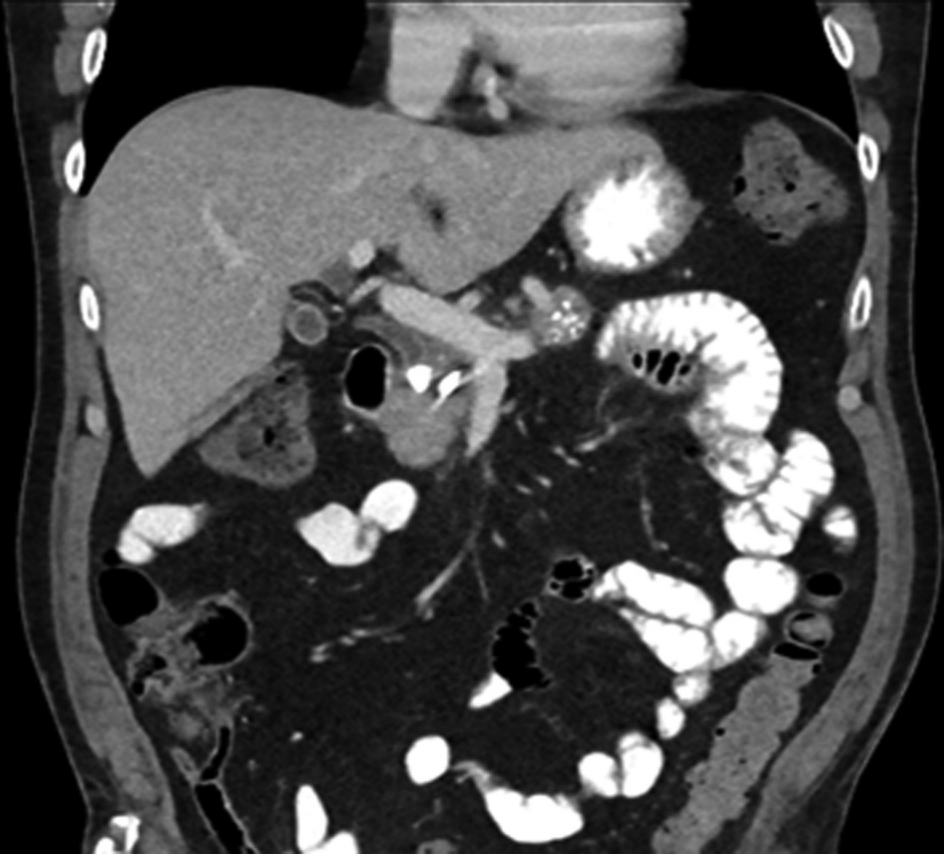
CT of the abdomen showing compression of the bile duct by the pancreatic stone.

A pancreaticobiliary fistula is considered a rare condition and is generally associated with pancreatic pseudocysts and acute necrotizing pancreatitis.^1^, ^2^, ^3^ Often, the fistula is managed by deployment of a plastic stent in the pancreatic duct, which may lead to closure of the fistula.^4^ Cholangitis due to pancreaticobiliary fistula has only been described in cases of intraductal papillary mucinous neoplasms or impacted pancreatic stones.^5^, ^6^, ^7^, ^8^, ^9^ Cholangitis due to a migrated pancreatic stone into the CBD, as was the case in our patient, is a very rare clinical presentation. In contrast to bile stones, which are usually cholesterol type stones and not radiopaque, pancreatic stones are usually visible on plain fluoroscopy images as focal calcifications, and appear as white calculi on endoscopic imaging. The removal of pancreatic stones often requires lithotripsy.

## Disclosure

*Dr. Poley and Bruno have consultancy/lecture agreements with Cook Endoscopy and Boston Scientific. All other authors disclosed no financial relationships relevant to this publication.*

## Supplementary data

Video 1 Electrohydraulic lithotripsy of migrated pancreatic stone in the bile duct.
